# Empowering personalized medicine: unleashing the potential of patient-derived explants in clinical practice

**DOI:** 10.17179/excli2023-6700

**Published:** 2024-01-10

**Authors:** Oliwia Piwocka, Wiktoria M. Suchorska, Katarzyna Kulcenty

**Affiliations:** 1 Department of Electroradiology, Poznan University of Medical Sciences, 61-701 Poznan, Poland; 2 Doctoral School, Poznan University of Medical Sciences, 61-701 Poznan, Poland; 3 Radiobiology Laboratory, Department of Medical Physics, Greater Poland Cancer Center, 61-866 Poznan, Poland

**Keywords:** patient-derived explants, personalized medicine, individual therapy, oncology

## Abstract

In recent decades, significant progress has been made in understanding the molecular characteristics of cancer and its microenvironment, leading to the development of life-saving treatments. However, patients often experience side effects from standard therapies, highlighting the need for personalized medicine. Personalized medicine aims to customize drug therapy and preventive care based on individual patients' specific requirements. The heterogeneity within tumors and among patients necessitates personalized medicine approaches. Patient-derived organoids (PDOs), xenografts (PDXs), and explants (PDEs) have emerged as valuable models for studying tumor behaviour and drug response. This paper aims to summarize the latest advancements in patient-derived explants, focusing on their potential utility in the clinic. Different methods for culturing PDEs, including the free-floating approach, the grid method, and sponge scaffolds, are discussed. These approaches provide opportunities for long-term viability, oxygen and nutrient supply, and maintenance of tissue integrity. Additionally, various solid tumor models using PDEs are highlighted, together with assays to study PDE viability, characteristics, and response to drug treatment.

## Introduction

For decades, researchers have focused on unraveling the molecular characteristics of cancer and its microenvironment, which led to the development of life-saving treatments for this severe disease. The effort is visible in statistics predicting decreasing trends for cancer mortality and incidence in colorectal, breast, prostate, leukemia, and stomach cancer (Malvezzi et al., 2023[28]). Despite various available therapies, patients experience side effects of their treatment. At this point, personalized therapy comes forward. Personalized medicine is a healthcare approach that customizes drug therapy or preventive care according to the specific requirements of individual patients. It involves utilizing an individual's molecular, genetic and epigenetic information while considering their preferences, beliefs, attitudes, knowledge, and social contexts (Strianese et al., 2020[46]). Current-day medical treatment has faced a significant drawback, as it has been primarily designed for the "average patient," assuming that all patients with the same disease have similar phenotypes/genotypes and should be treated uniformly. This "one-size-fits-all" approach often leads to varying effectiveness of treatments among different patients (Maier, 2019[26]). 

A tumor is a diversified ecosystem composed of stromal elements (blood and lymph vessels, extracellular matrix, immune cells, fibroblasts, endothelial cells) and tumor cells that all together compose the tumor microenvironment (TME) (Wei et al., 2020[49]). All these components may differ among tumors and patients, referred to as tumor heterogeneity. Tumors of the same histopathological subtype may exhibit distinct genotypes and phenotypes and display diverse biological behaviors. Cancer clinicians have also observed significant variations in tumor behavior among patients with the same tumor type and even within different tumor sites in the same patient. Heterogeneity is often evident through differential or mixed responses to therapy, which needs to be targeted by personalized medicine (Fisher et al., 2013[11]). For that purpose, scientists use patient-derived organoids (PDOs), -xenografts (PDXs), and -explants (PDEs). PDX are valuable models and are considered a high-standard tool for cancer research; however, they are expensive and have low throughput (Pettersen et al., 2023[38]). PDOs and PDEs are an alternative for PDX and follow the 3R rule: replacement, reduction and refinement in terms of animal usage (Hubrecht and Carter, 2019[17]). PDOs preserve genomic characteristics and are helpful in high-throughput screening, although they require enzymatic dissociation, which influences tissue integrity (Boucherit et al., 2020[4]; Wensink et al., 2021[50]; Zhou et al., 2021[53]). In 2018, Vlachogiannis and colleagues used PDOs in clinical trials and reported that they could predict patient responses to treatment, making them suitable for enrollment into personalized medicine (Vlachogiannis et al., 2018[48]). *Ex vivo* models are mostly explant models, in which a freshly resected tumor is used immediately for research. PDEs are gaining popularity in oncology studies since they do not depend on tissue reorganization and facilitate drug testing on entire tumor pieces (Powley et al., 2020[39]). Moreover, PDEs preserve features of the original tumor and maintain its stroma. The main drawback of explant culture is the limited ability for long-term culture, but this obstacle has been partially solved, i.e., in colorectal cancer by orbital shaking (Da Mata et al., 2021[9]).

Numerous hopeful treatments that demonstrate success in initial pre-clinical assessments eventually prove ineffective in clinical settings. This phenomenon might stem from established cell lines' limitations in accurately predicting outcomes of drug interventions in actual clinical conditions. Established cell lines lack tumor heterogeneity, and accumulating genetic alternations might impact the final results (Richter et al., 2021[41]). For instance, one of the Hsp90 inhibitors, 17-AAG, gave promising results in a pre-clinical setting, but it turned ineffective in patients during phase II of clinical trials (Heath et al., 2008[16]). Subsequent experiments comparing the activity of 17-AAG in cell lines and PDEs of prostate tumors were in line with clinical trial results (Nguyen et al., 2018[32]), concluding that PDEs might be a more suitable model for pre-clinical studies that spare funds and offers a faster response, which earlier was not observed until further phases of clinical trials.

This work aims to gather the latest accomplishments concerning patient-derived explants to disseminate explant culture and its potential utility in the clinic. Moreover, we summarized different approaches to culture PDEs and presented research on various solid tumor models of PDE.

## Platforms for PDE Culture

**Figure 1:**
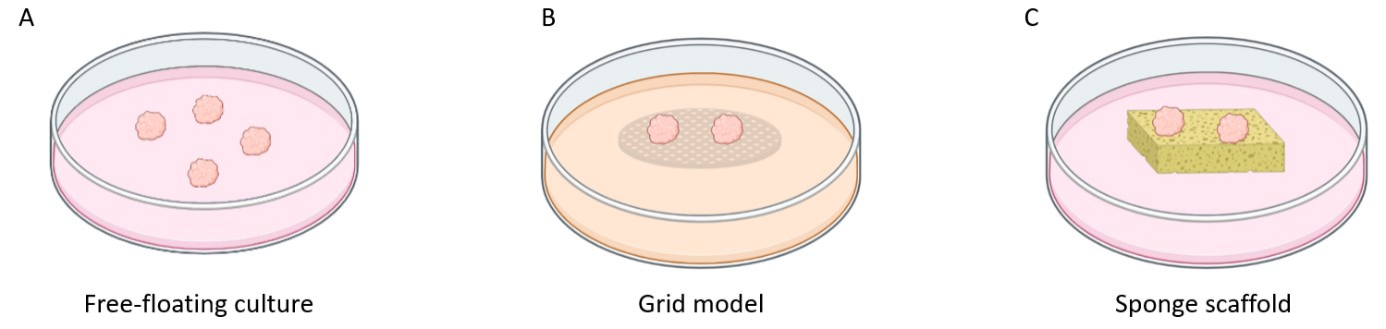
Three types of PDE models. A) Tissue fragments are entirely submerged in a medium and float freely, often combined with rotation on a shaker; B) Specimens are cultured on a metal grid, having contact with a medium and air, culture of tissue slices or cubes; C) Tissue fragments are placed on the sponge scaffold to provide simultaneous nutrient and oxygen supply. Figure created with BioRender.

Explants can be cultured through various methods (Figure 1(Fig. 1)). One approach is the submersion method, where the tissue is fully immersed in media within tissue culture dishes and floats freely. Another way is the grid method, where the tissue remains in contact with the media through a matrix or membrane supported by a plastic or metal grid. Additionally, researchers utilize gelatine or collagen sponges immersed in the media in the sponge method (Powley et al., 2020[39]).

### Free-floating approach

The free-floating or submerged method is the most accessible approach since it does not require additional inserts (Figure 1A(Fig. 1)). Evenly cut tumor pieces are immersed in the culture media and often placed on a rotation device. Free-floating cultures are applicable mainly for short-term explant cultivation due to the limited availability of oxygen, although rotation culture seems to resolve this issue (Wu et al., 2023[52]). Another drawback of the submerged method is the migration of epithelial cells toward the cut surface of the tissue and forming a capsule around it. To overcome migration, approaches such as grid and sponge culture were introduced to the research (Centenera et al., 2013[6]).

### Grid method

The grid method, also called an air-liquid interface (ALI) method, enables the culture of the whole explant and explant slices on the titanium grid immersed in a medium (Wu et al., 2023[52]) (Figure 1B(Fig. 1)). The backbone of this approach is simultaneous access to oxygen and nutrients by direct contact with media through a membrane supported by a metal frame (Powley et al., 2020[39]; Zhou et al., 2021[53]). In the case of explant slices culture, the essential step is to optimize the slice thickness depending on the type of tumor used. Too thin slices are delicate and may roll; too thick slices may encounter oxygen and nutrient deprivation (Nagaraj et al., 2018[30]). For lung cancer, 200 µm thick slices show the best viability (Nagaraj et al., 2018[30]); in prostate cancer, the most commonly used size is 300 µm (Kiviharju-af Hällström et al., 2007[21]; Ni et al., 2011[33]), while in pancreatic cancer, scientists used 350 µm thickness (Misra et al., 2019[29]). Davies and colleagues noticed necrotic tissue formation on explants cultured on a grid, which was significantly decreased in free-floating rotary culture (Davies et al., 2015[10]). Another issue with the usage of explant slices is the high heterogeneity among tumor specimens; thus, neighboring slices ought to be chosen as control and test samples to avoid differences in tissue composition. The grid method can also be combined with gentle rotation to moisten and aerate the explant (Nagaraj et al., 2018[30]).

### Sponge scaffolds

The sponge method is one of the most popular approaches for PDE, providing successful culture for breast, prostate, and pancreatic cancer (Kokkinos et al., 2021[22]). Sponge scaffolds are a commercially available, affordable platform to culture PDE that maintains hemostasis and is easy to use, which makes them applicable to many laboratories worldwide. A common problem faced in the free-floating PDE culture is cell outgrowth, which the sponge method also overcomes (Centenera et al., 2013[6]). The sponge scaffold allows nutrients to be absorbed by the explants through capillary action, eliminating the requirement to submerge the tissue in culture media, which could cause tissue degradation (Kokkinos et al., 2021[22]) (Figure 1C(Fig. 1)). The most commonly used sponge scaffolds are gelatine and collagen-based. Gelatine closely resembles the structural and functional properties of collagen. Thus, it often replaces collagen sponges in tissue culture (Bacero Bello et al., 2020[3]). 

Development of PDE culture on sponge scaffolds is simple and covers a few major steps. First, a 10 mm^3^ sponge must be soaked for 5-10 min and gently agitated in the same medium used for further cell culture. Agitation provides uniform permeabilization of the fluid, which is crucial for maintaining PDEs. Soaking the sponges for a prolonged time may cause their shrinkage. In the meantime, tumor specimens should be washed generously with PBS and cut into 1 mm^3 ^sections. The size of the dissected tissue is essential for preserving tissue morphology and integrity since too small or too large explants are subjected to necrosis (Centenera et al., 2013[6], 2022[7]). To summarize, PDE culture methods were compared in Table 1(Tab. 1).

### Advancements and limitations of PDE models

Recently, many advances have been made to improve PDE culture, especially its long-term viability. The most significant challenges in PDE culture include the absence of blood and lymph circulation, which suppresses the migration of immune cells into the tissue. The diffusion of drugs into the different regions of the tissue might face limitations, particularly for larger compounds (Misra et al., 2019[29]). Hypoxia inside an explant is a common problem causing necrosis (Nauta et al., 2014[31]). Researchers partially overcame this issue by culturing explants in rotation flasks without scaffolds. That approach extended the viability of explants up to 30 days in ovarian and colorectal PDEs by concurrently preserving molecular and histological features (Abreu et al., 2020[1]; Da Mata et al., 2021[9]). Another crucial aspect of PDE culture is the size of the explant, which is important in terms of drug and oxygen penetration. Studies show that the most suitable size is 1 mm^3^ for tissue parts and 200-300 µm thickness while working with tissue slices (Wu et al., 2023[52]). Some groups also apply additional devices and platforms to incorporate fluid flow, mimicking *in vivo* conditions. Liu and colleagues used inserts covered with a glass fibre scaffold and blood analogue applied under pressure to imitate perfusion and oxygen delivery, enhancing metabolic activity. This approach led to the development of perfused cell culture, which may be used for drug testing (Peng et al., 2023[36]). 

#### Limitations of in vitro culture

While the PDE model addresses several shortcomings present in current tissue-derived systems, it comes with its limitations. Notably, the PDE technique is *ex vivo* with finite culturing period, making it unsuitable for studying the de novo recruitment of immune cells (Shafi et al., 2019[45]). *In vitro* techniques typically lack the inclusion of a functional immune system, a vital component in the human body's response to diseases. The absence of immune cells and their dynamic interactions with tissues can compromise the physiological relevance of findings obtained from *in vitro* studies, particularly in the context of immunotherapy and infectious diseases. Usually, it is necessary to combine research results from various *in vitro* system to obtain reliable immunological response (Petrus-Reuer et al., 2021[37]). Traditional *in vitro* models often rely on static culture conditions that do not mimic the dynamic nature of physiological processes. Cells *in vivo* experience constant exposure to mechanical forces, shear stress, and fluid flow, all of which play pivotal roles in cellular behavior and can limit translation to clinical practice (Richter et al., 2021[41]). 

*In vitro* techniques have significantly advanced our understanding of human biology and disease, but it is imperative to recognize and address their limitations. Integrating these models into a broader framework that includes various systems and clinical validation will enhance their utility and contribute to more reliable and translatable research outcomes. By navigating these boundaries, researchers can unlock the full potential of *in vitro* techniques in shaping the future of biomedical research and healthcare.

## Explant Analysis

Once PDE culture is established, another essential step is assessing PDE size, viability, and molecular and histopathological characteristics. Afterward, endpoint analysis is executed to determine the effect of drugs or other factors on PDE.

PDE size is most commonly evaluated with brightfield microscopy and software for picture processing (Da Mata et al., 2021[9]). Various methods exist to measure PDE viability, including dye exclusion, membrane permeability approaches, and mitochondrial assays. One of the simplest methods is trypan blue, which stains dead cells blue (Kamiloglu et al., 2020[19]). In the case of cell suspensions, nonviable cells remain in the supernatant, and viable parts remain in the pellet that may be re-suspended to obtain only alive aliquots (Pettersen et al., 2023[38]). The membrane permeability approach includes live/dead assay utilizing fluorescein diacetate and propidium iodide that mark live and dead cells, respectively (Da Mata et al., 2021[9]). To indicate necrotic parts in the tumor sample, the JC-1 stain may be used to visualize healthy cells labeled in red (Kamiloglu et al., 2020[19]). Besides fluorescent microscopy methods, flow cytometry is also used to assess PDE viability. For this reason, dyes like Annexin V or MitoTracker can be used to evaluate apoptosis (Logue et al., 2009[24]). 

There are two approaches to assessing the response to the drug. The first option involves homogenizing the tissue; another way is to keep PDE intact and subject it to analysis for spatial approaches. Once the PDE is homogenized, techniques like mass spectrometry, transcriptomic, genomic, or metabolomic profiling, and flow cytometry can be utilized to determine biomarkers' expression (Powley et al., 2020[39]). Spatial techniques are promising methods enabling the analysis of intact specimens. Multiplexed immunofluorescence (mIF) provides complete information about TME and intercellular interactions, including immune cell infiltration. Moreover, mIF enables the quantification of single-cell surface markers and the placement of different cells in each tissue compartment (Sanchez et al., 2021[44]). mIF combined with mathematical and bioinformatical modeling is a powerful tool for studying TME and predicting drug responses (Parra, 2021[35]). Other approaches are spatial-omics techniques to study the entire, viable sample and its transcriptional, proteomic, or metabolomics pattern employing microarrays or microdissections (Williams et al., 2022[51]). Overall, the development of molecular biology methods may increase our understanding of PDE characteristics and contribute to the evolution of personalized medicine. 

## The Potential of PDEs in Clinical Practice

A significant part of cancer research is focused on finding a pre-clinical system suitable for estimating drug effectiveness. The shortage of sufficient pre-clinical models is the leading obstacle in elaborating innovative treatments, and it limits the ability to accurately predict individual responses to therapies (Ghosh et al., 2019[13]). 

The usage of PDX is currently one of the most reliable and promising settings; however, it also holds some drawbacks, i.e., the time of PDX development and poor success rates in some cancers. Taking this into account, PDE is a promising model since it bridges the gap between animal studies and clinical trials (Shafi et al., 2019[45]). PDEs enable response assessment in many tumors at a notably reduced expense compared to PDX under defined conditions, regardless of the toxicological factors encountered in animal models (Louandre et al., 2016[25]). The main difficulty in applying PDEs in clinics is their poor reproducibility. Nevertheless, PDEs might be suitable for trials focused on personalized medicine, such as basket, umbrella, and platform trials (Park et al., 2019[34]). The research utilizing explants may be compelling once combined with machine learning (ML), as presented by Majumder and colleagues, who used ML platform (CANScript technology) and PDEs to predict clinical outcomes for anti-cancer drugs with a sensitivity reaching 100 % in half of the patients (Majumder et al., 2015[27]). ML is applicable for analyzing multiple data types, i.e., images, laboratory records, demographic data, and creating a prognosis or possible treatment considering heterogeneity (Majumder et al., 2015[27]; Park et al., 2019[34]). 

**Figure 2:**
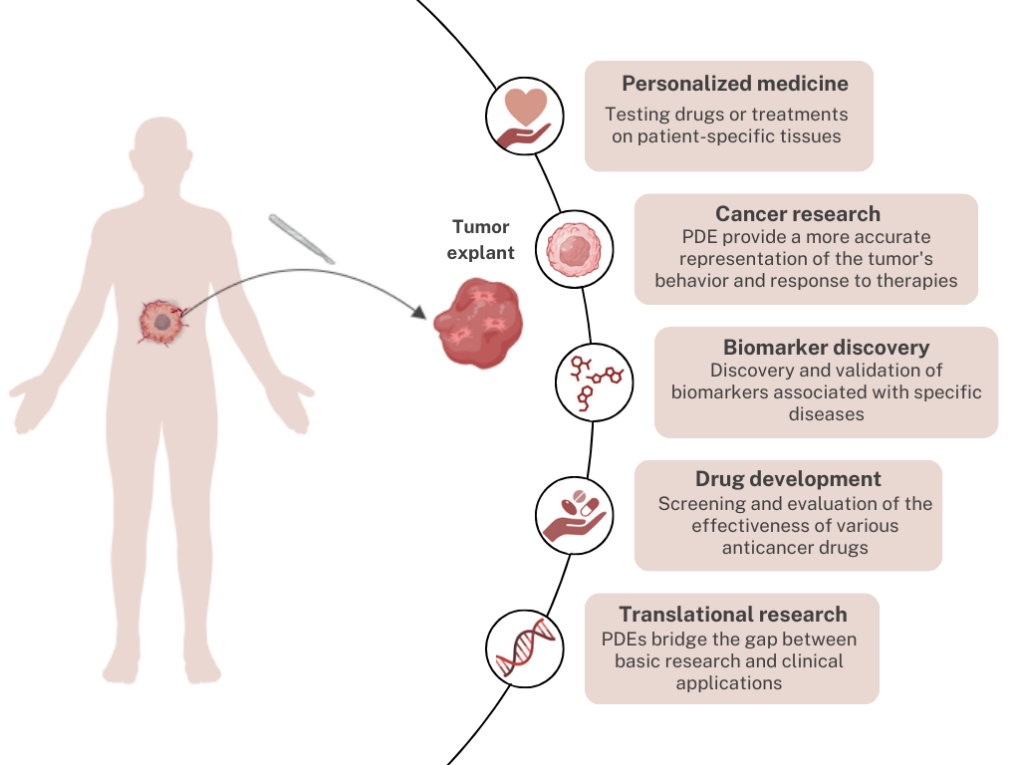
Applications of PDE models in research. Explants are the most commonly used in cancer research to test and validate various therapies considering personalized approach and tumor heterogeneity. PDEs are also valuable in biomarker discovery associated with different diseases, often leading to new drug development. Moreover, due to their unique properties, PDEs are bridging the gap between research and clinics.

Another difficulty in research translation to clinics is the deficiency of biomarkers monitoring individual patients' drug reactions. One of the reasons for poor biomarker availability comes from TME and transcriptomic heterogeneity. 3D *ex vivo *models retain TME with specific stromal interactions crucial for tumor growth and impact drug activity (Anderson and Simon 2020[2]; Nguyen et al., 2018[32]). Since explants maintain patients' heterogeneity and histopathology, they can potentially discover and validate biomarkers associated with diseases (Figure 2(Fig. 2)) (Powley et al., 2020[39]; Rodolfo et al., 2022[42]). Nguyen et al., combined PDEs with proteomic profiling to demonstrate that explants are clinically relevant tools and identified novel biomarkers for Hsp90 inhibitors in colorectal cancer (Nguyen et al., 2018[32]). 

PDEs are commonly used to assess compound impact on tissues, i.e., drugs, chemical agents, and inhibitors (Table 2(Tab. 2); References in Table 2: Abreu et al., 2020[1]; Centenera et al., 2018[5], 2022[7]; Collins et al., 2020[8]; García-Davis et al., 2019[12]; Gregory et al., 2020[14]; Kähkönen et al., 2021[18]; Karekla et al., 2017[20]; Kokkinos et al., 2021[22]; Lesko et al., 2021[23]; Misra et al., 2019[29]; Nguyen et al., 2018[32]; Peng et al., 2023[36]; Ricciardelli et al., 2018[40]; Saleh et al., 2020[43]). In breast cancer, PDEs were used to check the influence of benzophenone-3 in macrophage polarization and assess the phenotypic changes of macrophages by introducing interferons or interleukins (Gregory et al., 2020[14]). Another research group evaluated the effect of FGFR inhibitors in patient samples and showed results suggesting an increasing number of apoptotic cells (Kähkönen et al., 2021[18]). Saleh et al. examined the effect of immune checkpoint inhibitors and reported enhanced immune response and suppression of cancer-associated pathways (Saleh et al., 2020[43]). Preserved TME in explants enables examining the behavior of specific cells in the stroma. Rodolfo et al., analyzed an immune response to immune checkpoint inhibitors due to preserved tumor immune microenvironment (TIME) in explants of sarcoma and melanoma (Rodolfo et al., 2022[42]). Evaluation of cytotoxicity in preclinical studies is also a great advantage of PDEs. Patient tissues can be used to study the effect of novel agents, including laurinterol, which is a metabolite of red algae demonstrating anti-tumoral properties (García-Davis et al., 2019[12]), or to assess the response to chemotherapeutics, i.e. cisplatin in lung cancer (Karekla et al., 2017[20]) or carboplatin in ovarian cancer (Ricciardelli et al., 2018[40]). 

PDEs are helpful not only in cancer research but they have been applied in alcohol-induced liver damage research, where liver explants were subjected to ethanol (Hattersley et al., 2011[15]). Furthermore, explants may serve as a relevant platform to connect nanobiomaterials' (NBMs) studies to monitor the accumulation of nanoparticles (Tutty et al., 2022[47]).

## Conclusions

Patient-derived explants are a promising tool for personalized medicine and the study of tumor heterogeneity. By leveraging these models, researchers and clinicians can gain valuable insights into individualized drug responses, leading to more effective and tailored treatments for cancer patients. Short-term tumor explant cultures may not be applicable for studying the enduring consequences of processes like vascular involution induced by anti-angiogenic treatments. Instead, they are best suited for analyzing medical compounds that directly influence tumor cells by stimulating cell proliferation or inducing cancer cell death. PDE combined with advanced research methods has a chance to revolutionize current diagnostic procedures and personalized medicine.

## Declaration

### Authors' contribution

OP and KK were responsible for the conceptualisation of the manuscript. OP wrote the manuscript. KK and WMS provided editorial remarks and checked the manuscript. 

### Conflict of interest

The authors declare that they have no conflict of interest.

## Figures and Tables

**Table 1:**
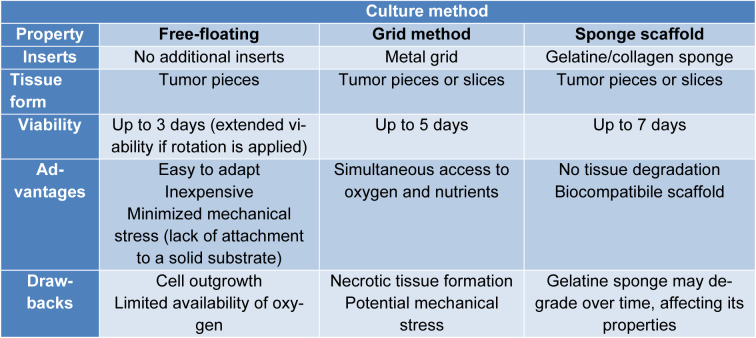
A summary of available methods of PDE culture, including their characteristic features, advantages and drawbacks. Choosing the most suitable method for patient-derived explant culture will depend on the specific research objectives, the cultured tissue type, and the desired level of experimental control and tissue preservation. Researchers often tailor their approach to achieve optimal study outcomes based on these factors.

**Table 2:**
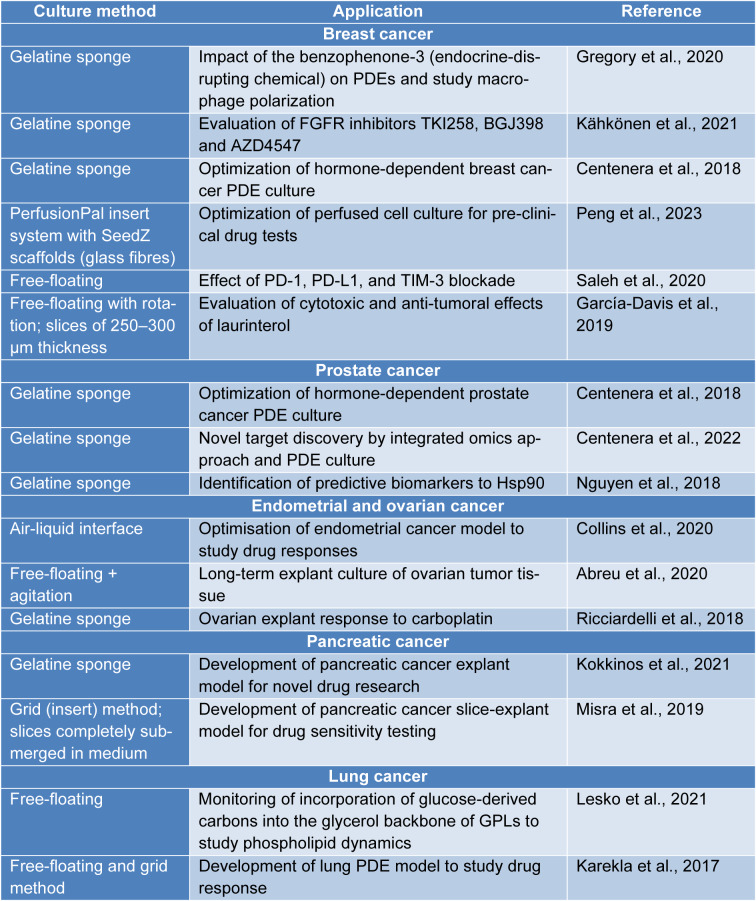
PDE culture models, methods and their application in some solid tumors
